# Chronic immobilisation stress ameliorates clinical score and neuroinflammation in a MOG-induced EAE in Dark Agouti rats: mechanisms implicated

**DOI:** 10.1186/1742-2094-7-60

**Published:** 2010-10-07

**Authors:** Beatriz G Pérez-Nievas, Borja García-Bueno, José LM Madrigal, Juan C Leza

**Affiliations:** 1 Department of Pharmacology, Faculty Medicine, University Complutense, Centro de Investigación Biomédica en red de Salud Mental (CIBERSA), Granada, Spain; 2 Instituto de Investigación Sanitaria Hospital 12 de Octubre, Madrid 28040, Spain

## Abstract

**Background:**

Multiple sclerosis (MS) is the endpoint of a complex and still poorly understood process which results in inflammation, demyelination and axonal and neuronal degeneration. Since the first description of MS, psychological stress has been suggested to be one of the trigger factors in the onset and/or relapse of symptoms. However, data from animal models of MS, such as experimental autoimmune encephalomyelitis (EAE) are inconsistent and the effect of stress on EAE onset and severity depends on duration and time of application of the stress protocol and the underlying mechanisms.

**Methods:**

Dark Agouti rats were inoculated with MOG/CFA to induce EAE, and an immobilisation stress protocol with two different durations (12 and 21 days, starting at the moment of MOG-inoculation) was applied in order to analyse the effect of stress on disease onset and neuroinflammation.

**Results:**

Twelve days of stress exposure increased EAE clinical score in Dark Agouti rats. In addition, these animals presented higher levels of MMP-9 and proinflammatory PGE_2 _in spinal cord. In contrast, animals chronically exposed to stress (21 days) showed a significantly lower incidence of EAE clinical signs and reduced myelin loss, leukocyte infiltration and accumulation of inflammatory/oxidative mediators in spinal cord. Interestingly, chronically stressed animals showed a parallel increase in levels of the anti-inflammatory prostaglandin 15d-PGJ_2_, the main endogenous agonist of PPARγ.

**Conclusions:**

Our results demonstrate that, depending on duration, stress exposure elicits opposite effects on PGE_2_/15d-PGJ_2 _ratios in spinal cord of EAE-induced Dark Agouti rats. Further studies are needed to elucidate if these changes in prostaglandin balance are sufficient to mediate the differences in clinical score and inflammation here reported, and to establish the potential utility of pharmacological intervention in MS directed toward anti-inflammatory pathways.

## Background

Multiple sclerosis (MS) is a chronic, disabling disease of the central nervous system (CNS), affecting more than 2 million people worldwide. It has an autoimmune component and is characterized by chronic neuroinflammation with lymphocyte infiltration into the CNS, myelin loss, gliosis, various degrees of axonal and oligodendrocyte pathology and progressive neurological dysfunction [1].

Although the entire picture is still not clear, there are several factors linked to higher risk of developing the disease: 1) Genetics may play an important role and several alleles have been identified as predisposing factors for the disease, most of them related to the human leukocyte antigen (HLA) system [2]; 2) the clinical and pathological features of MS implicate viral infections as either cofactors in its aetiology or as triggers for relapse (especially Herpes and Epstein Barr viruses) [3,4]; 3) MS is more prevalent in women than in men, suggesting importance of hormones in its pathophysiology [5]; 4) since the first description of MS, psychological stress has been implicated in triggering exacerbations, and some meta-analyses suggest a positive relationship between stressful events and higher risk for relapse [6] and increased risk of new gadolinium-enhancing (Gd+) brain lesions [7]. However, while studies of animal models have potential to explain the pathophysiological basis of these interactions, data from these animal models are inconsistent (reviewed in [8]): in general, acute stress before inoculation increases the severity of the disease [9,10], while chronic stress has been reported to have a protective role, but only when applied prior to induction of EAE [11-13]. However, data obtained from the Theiler's murine encephalomyelitis virus (TMEV) model are sometimes contradictory and still inconclusive [14-17]. On the other hand, Whitacre et al., (1998) have shown that stress exposure after induction of relapsing EAE has no protective effect [13].

The general response to stress includes release of glucocorticoids as a consequence of activation of the hypothalamic-pituitary-adrenal (HPA) axis. Glucocorticoids are considered to be anti-inflammatory, immunosuppressive and immunomodulatory in the periphery under normal conditions. The mechanisms underlying these peripheral effects include inhibition of lymphocyte proliferation and dendritic cell maturation, as well as apoptosis of basophils, eosinophils and T-cells [18]. However, in recent years, this classic view that glucocorticoids are universally anti-inflammatory has been challenged, mainly in the CNS [19]. In particular, in the brain, glucocorticoids are not uniformly anti-inflammatory, and can even have pro-inflammatory actions on functions such as cell extravasation and migration and transcription factor activity [20], when released at supraphysiological levels.

Indeed, exposure to restraint stress induces the release of proinflammatory cytokines such as tumor necrosis factor-α (TNFα) in the CNS, which activates inflammatory nuclear transcription factor κB- (NFκB) dependent pathways inducing expression of inducible NO synthase (iNOS) and of the inducible form of cyclooxygenase (COX2), among other inflammatory mediators [21]. The result of these effects is the accumulation of proinflammatory, oxidative and nitrosative mediators (i.e. prostaglandin E_2 _(PGE_2_) and reactive oxidant species, such as nitric oxide (NO) or peroxinitrite anion (ONOO^-^)). Some of these, being free radicals or highly reactive compounds, can attack membrane phospholipids and cause cell damage by lipid peroxidation [22].

Noticeable anti-inflammatory pathways are also activated in brain in response to immobilisation stress, constituting a possible endogenous mechanism of defence against excessive inflammation. One of the mechanisms that enables "central neurogenic protection" [23] is activation of a cyclooxygenase subpathway, which leads to an increase in levels of the anti-inflammatory prostaglandin 15deoxi-prostaglandin J_2 _(15d-PGJ_2_) and he activation of its main cellular target, peroxisome proliferator-activated receptor gamma, PPARγ [24,25].

PPARγ and its agonists regulate cerebral physiology and have been proposed as potential therapeutic targets for the treatment of several pathological conditions related with neuroinflammation within CNS; i.e. PPARγ agonists exert a broad spectrum of protective effects in several animal models of neurological and cardiovascular diseases (reviewed in [26]). In the case of EAE, PPARγ activation by both endogenous (15d-PGJ_2_) and synthetic (thiazolidinediones) agonists ameliorates the pathophysiology of the disease, decreasing the clinical symptoms by reducing inflammation [27-32].

Taking into account the above-described opposite effects of stress exposure on the course of EAE, depending on the type of stress and the moment of its application, we submitted EAE-induced Dark Agouti rats to immobilisation stress and studied stress-induced changes in EAE clinical signs, myelin loss, disruption of brain blood barrier (BBB), leukocyte infiltration and accumulation of inflammatory/oxidative mediators in the spinal cord at two different time points: 12 and 21 days.

## Methods

### Animals

Forty eight young-adult (12-week old) male Dark Agouti rats (DA/OlaHsd from Harlan Iberica) weighing 225-250 g were used. All experimental protocols followed the guidelines of the Animal Welfare Committee of the Universidad Complutense according to European legislation (2003/65/EC). Rats were housed individually under standard conditions of temperature and humidity and a 12-h light/dark cycle (lights on at 0800 h) with free access to food and water. All animals were maintained under constant conditions for at least 7 days prior to experiment.

### Induction of EAE

Several different EAE models have been developed, which differ in the grade and time of appearance of immunological reactions and inflammatory processes within the CNS. For this study, the EAE-inductor agent chosen was myelin oligodendrocyte glycoprotein (MOG) which is a unique myelin autoantigen as it is able to induce not only an encephalitogenic T-cell response in susceptible species, but also a demyelinating autoantibody response [33]. This specific model shows several similarities to MS, such as a relapsing-remitting disease course, demyelination, and axonal degeneration [34]. Rats were immunized subcutaneously at the base of the tail with 150 μg of rat MOG peptide (fragment 35-55) (Sigma, Madrid, Spain) emulsified in Freund's adjuvant (Sigma) supplemented with 400 μg of heat-inactivated *Mycobacterium tuberculosis *(H37Ra, (DIFCO, Detroit, MI, USA)) to make complete Freund's adjuvant (CFA) in a total volume of 100 μL per rat. This EAE model mimics the relapsing-remitting disease course, which is present in the majority of MS patients [35].

Animals were weighed and scored daily for signs of EAE according to the following scale: 0, no disease; 1, tail paralysis; 2, hind limb weakness; 3, hind limb paralysis; 4, hind limb paralysis plus forelimb weakness; 5, moribund or dead.

### Stress model

**Figure 1:**
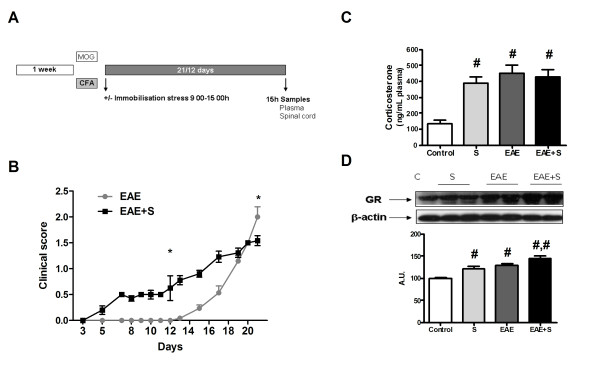
**Chronic stress ameliorates MOG-induced EAE in DA rats**. **A **Experimental design: one week after habituation of animals they were inoculated with MOG/CFA (EAE and EAE+S groups), only with CFA (Control and S groups) and submitted (S and EAE+S groups) or not (Control and EAE groups) to stress for 21 or 12 days depending on the experiment. **B **Clinical score in EAE-induced animals submitted or not to stress according to this score: 0, no disease; 1, tail paralysis; 2, hind limb weakness; 3, hind limb paralysis; 4, hind limb paralysis plus forelimb weakness; 5, moribund or dead. Clinical signs appeared earlier in the EAE+S group, but at day 21 the clinical score for the EAE group was 1.88 vs 1.54 in the EAE+S group. **C **Stress and EAE induced an increase in plasma corticosterone levels. **D **GR expression in spinal cord was increased by EAE and stress, and the EAE+S group showed the highest expression. # p < 0,05 vs Control; ## p < 0,01 vs Control; *p < 0,05 vs EAE one way ANOVA test followed by a Newman-Keuls (MOG: myelin oligodendrocyte glycoprotein; CFA: Complete Freunds Adjuvant; EAE: Experimental Autoimmune Encephalomyelitis).

Rats were exposed to 6 h of immobilisation stress between 0900 and 1500 h in the animal homeroom for 21 or 12 days in two different sets of experiments starting the same day of the inoculation with MOG in the case of the EAE and stress group of animals. Another group of animals was submitted to stress (21 or 12 days depending on the experiment) and received only vehicle. Animals from the control group were not immobilised and received only vehicle. EAE animals were not stressed after inoculation with MOG/CFA. Experimental groups were: (1) control (Control n = 6); (2) stress for 21 or 12 days (S n = 6); (3) MOG/CFA-inoculated (EAE n = 6); and (4) inoculated with MOG/CFA and submitted to stress for 21 or 12 days (EAE+S n = 6). The experimental design is shown in figure 1A. Restraint was performed using a plastic rodent restrainer that allowed for a close fit to rats during 6 h [36] in their home cages to minimise the effects of the obligate social isolation when placed in the individual restrainer. Animals were sacrificed, still in the restrainer, immediately after restraint using sodium pentobarbital (320 mg/Kg i.p.). Blood for plasma determinations was collected by cardiac puncture and anticoagulated in the presence of trisodium citrate (3.15% w: v, 1 vol citrate per 9 vol blood). Spinal cord was excised from the backbone and sections from the thoracic and lumbar spinal cord were extracted and snap frozen at -80°C until assayed.

### Corticosterone levels

Corticosterone was measured at the end of the experiment (day 21 or 12) in plasma from blood samples obtained by cardiac puncture at 1500 h. After blood centrifugation at 1,000 × g for 15 min, all plasma samples were stored at -80°C before assay using a commercially available kit based on RIA with ^125^I-labeled rat corticosterone (Siemens Healthcare Diagnostics S.L, Barcelona, Spain). A gamma counter (Wallac Wizard 1470, Perkin Elmer, Waltham, MA, USA) was used to measure radioactivity of the samples. The detection limit for the kit is 5.7 ng/mL, the intra-assay coefficient of variation is 4.3% and the interassay coefficient of variation is 5.8%.

### Western blot analysis

After determination and adjusting protein levels, homogenates of spinal cord tissue, once centrifuged (12000 g, 20 min at 4°C) were mixed with Laemmli sample buffer (Bio Rad, Hercules, CA, USA) (SDS 10%, distilled H2O, glycerol 50%, Tris HCl 1M pH 6,8, dithiotreitol and blue bromophenol) with beta mercapthoethanol (50 μL per mL of Laemmli) and 20 μL (2 μg/μL) were loaded into an electrophoresis gel. Once separated by molecular weight, proteins from the gels were blotted onto a polyvinylidene difluoride membrane (Millipore, Bedford, MA, USA) or nitrocellulose membrane (Amersham Biosciences Europe GmbH, Friburg, Germany) by semidry transfer system (Bio Rad) and they were incubated with specific antibodies: (a) glucocorticoid receptor (GR) antibody from Santa Cruz Biotechnology (Santa Cruz, CA, USA) raised against an epitope mapping at the N-terminus of GR α of mouse origin; (b) matrix metalloprotease-9 (MMP-9) rabbit polyclonal antibody from Chemicon generated using *E. Coli*-expressed active rat 92 kDa type IV collagenase (catalytic domain) as immunogen. The dilution of the antibody was 1:1000 in TBS-Tween; (c) myelin basic protein (MBP) rabbit polyclonal antibody from Santa Cruz Biotechnology (Santa Cruz, CA, USA) raised against amino acids 134-304 (deletion 193-218) of MBP of human origin in a dilution of 1:1000 in TBS-tween; (d) NOS-2 rabbit polyclonal antibody from Santa Cruz Biotechnology raised against a peptide mapping at the amino terminus of NOS-2 of human origin in a dilution of 1:1000 in TBS-Tween; (e) COX-2 goat polyclonal antibody from Santa Cruz Biotechnology raised against a peptide mapping at the C-terminus of COX-2 of human origin in a dilution of 1:750 in 5% skim milk in TBS-Tween; and (f) lipocalin PGDS rabbit polyclonal antibody from Cayman (Cayman Chemical Europe, Tallinn, Estonia) which recognizes human lipocalin-type PGD synthase amino acids 30-41 (VQPNFQPDKFLG) in a dilution of 1: 500 in 5% skim milk in TBS-Tween. As a loading control, mouse monoclonal β-actin antibody from Sigma was used. The immunogen was a slightly modified β-cytoplasmic actin N-terminal peptide, Ac-Asp-Asp-Asp-Ile-Ala-Ala-Leu-Val-Ile-Asp-Asn-Gly-Ser-Gly-Lys, conjugated to KLH. Proteins recognised by the antibody were visualised on X-ray film by chemiluminiscence (ECL) following the manufacturer's instructions (Amersham lberica). Autoradiographs were quantified by densitometry (program Image J, NIH), and several time expositions were analysed to ensure the linearity of the band intensities.

### Myeloperoxidase (MPO) activity assay

Immediately after sacrifice and dissection, spinal cord samples were minced on ice and homogenized (glass/glass) in 0.5% hexadecyltrimethyl ammonium bromide, 0.5% Nonidet P40 (Roche Farma, Madrid, Spain) in 20 mM phosphate buffer, pH 6.0. The homogenates were then centrifuged for 20 min at 12,000 g. Tissue levels of MPO activity were determined in supernatants using hydrogen peroxide as a substrate for the enzyme. A unit of MPO activity was defined as that converting 1 μmol of hydrogen peroxide to water in 1 min at 40°C [37].

### Lipid peroxidation

Lipid peroxidation rate was measured in spinal cord homogenates by the thiobarbituric acid test for malonildialdehyde (MDA) following the method described by Das and Ratty (1987) with some modifications. Spinal cord fragments were sonicated in 10 vol 50 mM phosphate buffer and deproteinised with 40% trichloroacetic acid and 5 M HCl, followed by the addition of 2% (w/v) thiobarbituric acid in 0.5 M NaOH. The reaction mixture was heated in a water bath at 90°C for 15 min and centrifuged at 12,000×g for 10 min. The pink chromogen was measured at 532 nm in a Beckman DU-7500 spectrophotometer (Beckman Coulter, Madrid, Spain). The results are expressed as nanomol per milligram of protein.

### Prostaglandin levels

Spinal cord PGE_2 _and 15d-PGJ_2 _levels were determined using enzyme immunoassay (EIA) kits (Cayman and DRG Diagnostics, Marburg, Germany). Samples were purified using Amprep^© ^minicolums (Amersham). After homogenising spinal cord fragments by sonication (Branson sonifier 250, American Lab Trading, East Lyme, CT, USA) in PBS with Tris HCl, NaCl, glycerol, 1% Triton X100 and protease inhibitor cocktail, the fraction needed for EIA was isolated and prostaglandin quantification was carried out following the manufacturer's instructions.

### Protein assay

Protein levels were measured using Bradford's method based on the principle of protein-dye binding (Bradford, 1976).

### Chemicals and statistical analyses

Unless otherwise stated, the chemicals were obtained from Sigma. Data in text and figures are expressed as mean ± SEM. Differences in biochemical parameters between groups were tested by the one way ANOVA test followed by a Newman-Keuls test once normality of distribution was assed and due to the sample size. A p value < 0.05 was considered statistically significant. As there are actually two sets of experiments, it is not acceptable to perform a two-way ANOVA and introduce time of stress as a factor.

## Results

### Effect of chronic stress on EAE

#### Effect of stress on EAE onset and severity

As it can be observed in figure 1B, clinical signs appeared earlier (days 5-8) in the EAE+S group than in the EAE group (days 12-15). However, after 21 days of experiment, animals from the EAE group showed higher clinical score than those submitted to 6 hours of daily immobilisation stress (EAE+S group) (1.88 vs 1.54, p < 0.05; according to following clinical score: 0, no disease; 1. tail paralysis; 2. hind limb weakness; 3. hind limb paralysis; 4. hind limb paralysis plus forelimb weakness; 5. moribund or dead).

These data indicate that the 21-day-restraint animals exhibit a significantly lower incidence of EAE clinical signs, lower mean clinical score and lower cumulative score. Thus prolonged restraint stress has a long-term suppressive effect on disease, resulting in a significant reduction in clinical score.

#### Effect of chronic stress and EAE on plasma corticosterone levels

Since glucocorticoids have been closely associated with the onset and severity of EAE, we measured corticosterone plasma levels in the different groups. As expected, EAE induced a significant increase in plasma corticosterone levels on day 21 after inoculation with MOG/CFA (EAE: 451,1 ± 53,37 ng/mL vs control: 134,7 ± 21,16 ng/mL). Chronic immobilisation stress had a similar effect on corticosterone levels (S: 388 ± 42,59 ng/mL). However, the increase in plasma corticosterone in the group of animals submitted to stress and inoculated with MOG/CFA was not statistically different from the other groups (EAE+S: 425,8 ± 49,24 ng/mL) (figure 1C, F: 23,08, dF: 33). Analysis of another indicator of HPA axis activity and regulation, glucocorticoid receptor (GR) expression, showed an increase in all groups of animals compared with their controls, 21 days after the onset of experiment (figure 1D; F: 14,74, dF: 7), indicating a normal regulation of the HPA axis.

#### Effect of chronic stress and EAE on BBB permeability, leukocyte infiltration and myelin levels in spinal cord

**Figure 2:**
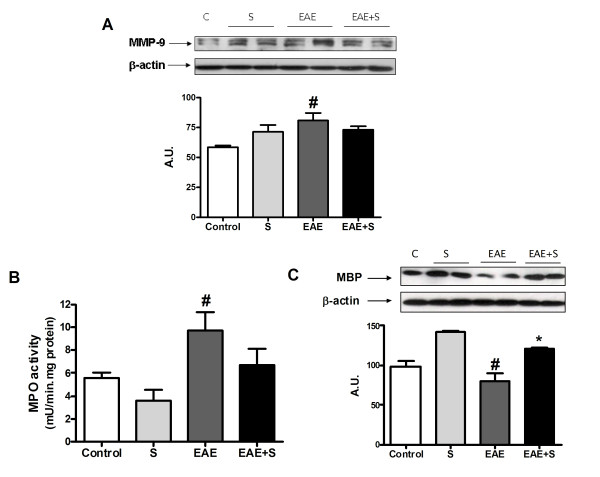
**Chronic stress reduces demyelination in MOG-EAE, MMP-9 expression and leukocyte infiltration**. **A **Analysis of Matrix metalloprotease 9 (MMP-9) expression in spinal cord by western blot and densitometric analysis of the band showed higher BBB disruption in the EAE group than in the EAE+S group. (β actin expression was used as internal control) **B **MPO activity in spinal cord was increased in spinal cord of EAE animals **C **Levels of MBP in spinal cord measured by western blot showed demyelination in the EAE group. β-Actin expression was used as an internal control and densitometric analysis of the band of interest is shown below. *p < 0,05 vs EAE one way ANOVA test followed by a Newman-Keuls.

MMP-9 up-regulation has been associated with blood-brain barrier (BBB) dysfunction in multiple neuropathological scenarios [38,39]. Western blot studies showed that animals stressed for 21 days after inoculation with MOG showed lower MMP-9 expression in spinal cord than animals only inoculated with MOG (figure 2A; F: 2,332, dF: 13). This observation suggests a possible higher degree of BBB disruption in EAE-induced animals than in EAE-induced animals submitted to chronic stress.

Higher BBB disruption led to an increase in leukocyte infiltration, measured by myeloperoxidase (MPO; H_2_O_2 _oxidoreductase) activity in spinal cord. MPO is an enzyme found in the azurophilic granules of mammalian neutrophils and also identified in human monocytes, and its activity has already been taken as a marker of leukocyte infiltration [40]. The EAE group showed enhanced MPO activity compared to the control group (9,66 ± 1,59 vs 5,59 ± 0,42 mU/min.mg protein respectively: see figure 2B; F: 6,708, dF: 15) while no increase in MPO activity was observed in the EAE group submitted to chronic stress, indicating leukocyte infiltration in spinal cord of the EAE group, but not of the EAE+S group, which could be directly related to the effects on MMP-9 expression above mentioned.

Parallel to MMP-9 upregulation and MPO overactivity, the demyelinating lesion, assessed by myelin basic protein (MBP) levels in spinal cord, was higher in the EAE+S group than in EAE: Western blot analysis in figure 2C showed a consistent decrease in MBP levels in spinal cord homogenates from the EAE group 21 days after MOG inoculation, but not in EAE-induced animals submitted to stress (EAE+S) (F: 10,17, dF: 13).

#### Oxidative parameters in spinal cord after EAE and chronic stress protocol

**Figure 3:**
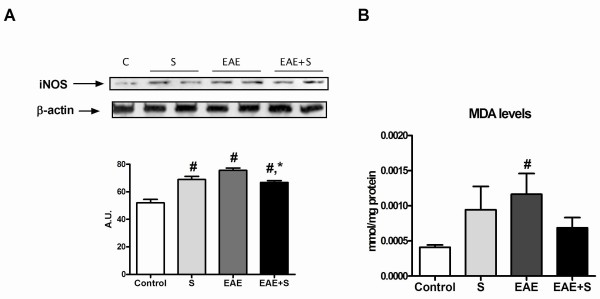
**Chronic stress reduces the EAE-induced increase in inflammatory/oxidative status**. **A **Western blot analysis revealed that iNOS expression in spinal cord homogenates was higher in EAE than in the EAE+S group; β-actin was used as an internal control and densitometric analysis of the band of interest is shown. **B **Lipid peroxidation rate measured by levels of MDA was also higher in spinal cord of the EAE group compared to the EAE+S group. *p < 0,05 vs EAE;. # p < 0,05 vs Control, one way ANOVA test followed by a Newman-Keuls.

Our next studies were aimed at elucidating the effects of stress exposure on the neuroinflammatory/oxidative process elicited by EAE in spinal cord of Dark Agouti rats. Both EAE and chronic stress induced an increase in iNOS expression compared with the control group in spinal cord homogenates, as measured by western blot. However, animals from the EAE group showed higher iNOS expression compared to the EAE+S group (figure 3A and densitometric analysis of the band of interest: F: 24,54, dF: 16). This fact correlated with a higher lipid peroxidation rate (determined by measuring levels of MDA, a product derived from membrane phospholipid attack) observed in spinal cord homogenates of the EAE group (figure 3B) compared with EAE+S (F: 92,15, dF: 13)

#### Balance of inflammatory/anti-inflammatory mediators in spinal cord after EAE and stress: COX-2 and prostaglandins

**Figure 4:**
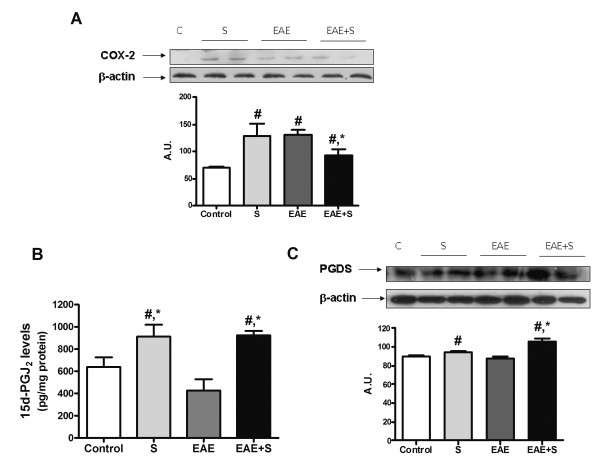
**The COX2 pathway is shifted towards anti-inflammatory mediators in EAE+S group**. **A **COX2 expression measured by western blot is lower in spinal cord of animals from the EAE+S group compared to the EAE group. **B **Chronic stress increased levels of the anti-inflammatory prostaglandin 15d-PGJ_2 _in spinal cord. **C**. Expression of lipocalin PGD synthase, the final enzyme responsible for synthesis of 15d-PGJ_2 _was also higher in spinal cord of the EAE+S group. β-Actin was used as internal control and densitometric analysis of the band of interest is shown below. *p < 0,05 vs EAE;. # p < 0,05 vs Control, one way ANOVA test followed by a Newman-Keuls

As in the case of iNOS expression, both stress and immunization with MOG/CFA induced an increase in the expression of the inducible isoform of cyclooxygenase (COX2) in spinal cord homogenates after 21 days. Exposure to chronic stress reduced this increase of expression, as can be observed in figure 4A (group EAE+S compared to EAE: F: 5,032, dF: 7). However, there were no significant differences in spinal cord levels of proinflammatory PGE_2_, which is one of the products derived from the activity of COX2, in none of the groups studied (data not shown).

Conversely, as it can be observed in figure 4B, there were differences in the levels of another COX2-derived product: the anti-inflammatory prostaglandin 15d-PGJ_2_. In fact, stress induced an increase in 15d-PGJ_2 _levels (912,8 vs 638,7 pg/mg protein in control group). This increase could also be observed in the EAE+S group when compared to the EAE group (924,1 vs 427,7 pg/mg protein: F: 6,897; dF: 26). These higher levels of anti-inflammatory prostaglandin correlated with higher expression of its specific enzymatic source, lipocalin prostaglandin D synthase (PGDS) in the S and EAE+S groups (western blot analysis in figure 4C with densitometric analysis of the band, F: 19,88, dF: 7). Twenty-one days after EAE induction, stress may induce a shift of anti-inflammatory/proinflammatory prostaglandin balance towards the former, which could have beneficial effects.

#### Effect of subchronic stress (12 days) on EAE

**Figure 5:**
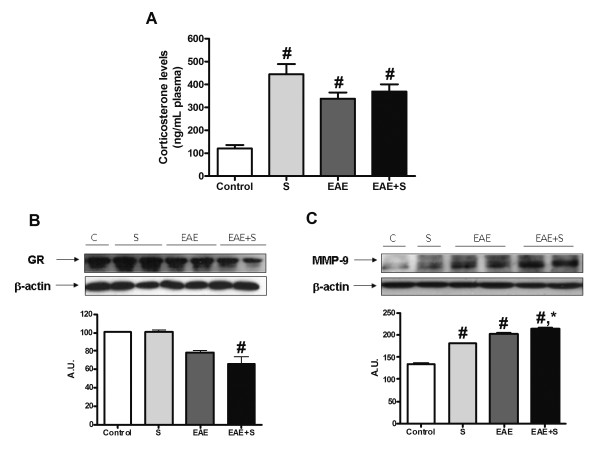
**Subchronic stress increases EAE-induced MMP-9 expression**. **A **Again EAE and stress induced an increase in plasma corticosterone but expression of GR in spinal cord measured by western blot revealed that the EAE+S group showed lower levels, indicating a failure in HPA regulation (**B**). **C **MMP-9 expression in spinal cord by western blot showed an opposite pattern to that at day 21, with higher levels in the EAE+S group than in the EAE group. β-Actin expression was used as an internal control and densitometric analysis of the band is shown below. *p < 0,05 vs EAE;.# p < 0,05 vs Control, one way ANOVA test followed by a Newman-Keuls.

After the evaluation of the chronic stress results, a new set of experiments was carried out to study the animals on day 12, when the EAE+S group shows clinical signs but the EAE group still does not (figure 1B). Clinical signs were scored daily, as previously, and the same pattern as in the other experiment was shown (figure is not included to make reading easier). The EAE, S AND EAE+S groups show increases in plasma corticosterone levels similar to that seen after 21 days, with no significant differences between them (figure 5A, F: 40,22, dF:41). Interestingly, expression of GR in spinal cord showed an opposite pattern to that in the 21-day experiment, showing a lower expression in the EAE+S group compared to the control group, which revealed differences in HPA axis regulatory mechanisms (figure 5B: F: 21,41, dF:7).

**Figure 6:**
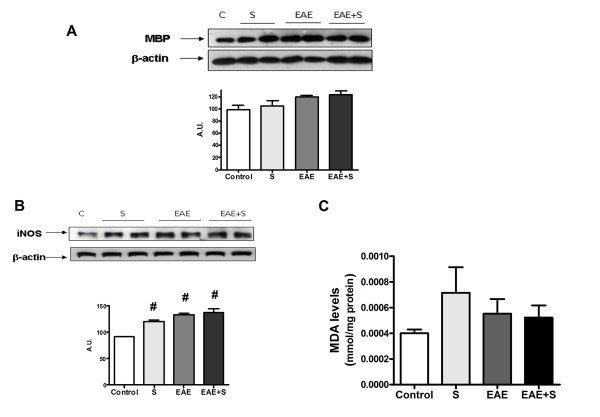
**No demyelination is observed at day 12 of the experiment and there are no differences in oxidative status in spinal cord of the EAE and EAE+S groups**. **A **Levels of MBP protein in spinal cord measured by western blot revealed no differences between the groups. **B **iNOS expression in spinal cord is increased by EAE and stress, but there are no statistically significant differences between the EAE+S and EAE groups. β-Actin expression was used as an internal control and densitometric analysis of the band is shown below. **C **Measurement of lipid peroxidation rate in spinal cord showed no differences between groups. *p < 0,05 vs EAE;. # p < 0,05 vs Control, one way ANOVA test followed by a Newman-Keuls.

**Figure 7:**
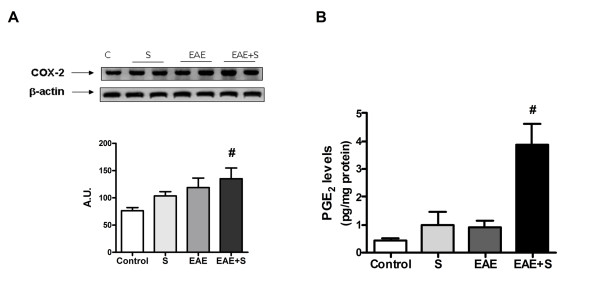
**Subchronic stress enhances proinflammatory PGE**_**2 **_**in EAE-induced DA rats**. **A **COX2 expression was higher in spinal cord of the EAE+S group at day 12, showing an opposite pattern to that at day 21, and (**B**) was correlated with higher levels of proinflammatory prostaglandin in spinal cord. β-Actin expression was used as an internal control and densitometric analysis of the band is shown below *p < 0,05 vs EAE;. # p < 0,05 vs Control, one way ANOVA test followed by a Newman-Keuls.

At day 12, MMP-9 expression was higher in the EAE+S group compared to the EAE group (figure 5C: F:405,1, dF:7), suggesting that stress could be enhancing EAE-induced BBB dysfunction, in contrast to what was observed on day 21. However, at this time point, no demyelination was observed (figure 6A) and no differences in levels of MBP in spinal cord homogenates measured by western blot were observed. EAE and stress increase expression of iNOS (figure 6B: F: 26,53, dF: 7) and, although there is a trend toward the iNOS increase in expression to be higher in the EAE+S group, there are no statistical differences when compared to the EAE group. No differences in lipid peroxidation in spinal cord rate were found for any of the groups (figure 6C: levels of MDA). However, the increase in COX2 expression was higher in the EAE+S group compared to EAE, in contrast to what was observed after 21 days (figure 7A). Moreover, at 12 days PGE_2 _levels were higher in the EAE+S group compared with the other groups (3,887 pg/mg protein vs 0,4199 in control group, 0,9794 in stress group and 0.9165 in EAE group: F: 11,97, dF: 14, see figure 7B) and no differences were found between groups in anti-15d-PGJ_2 _levels (data not shown), suggesting a proinflammatory balance of prostaglandins for this time-point of experiment that could account for the increase in clinical score reported.

## Discussion

The precise mechanisms through which stress regulates susceptibility and severity of autoimmune diseases have been extensively investigated. The biological impact of stress is exerted via a feedback regulatory loop within a network consisting of the central nervous system (CNS), the endocrine system and the immune system. It has already been established that effects of stress on immune function are highly dependent on the type and duration of the stressor [41]. Consequently, the effects of stress on EAE development depend not only on type, timing and duration of the stressor, but also on rat strain and the chosen EAE model [6]. Repeated moderate stressors may act by suppressing signs when given before disease induction, whereas acute severe stressors may enhance the progression of disease after its induction [8]. The possibility that chronic stress reduces the immunization response to MOG in the periphery, negatively modulating CNS inflammatory responses, might be a confounding factor in our study. However quantification of plasma levels of IFNγ, as a main peripheral immune parameter in EAE, showed no differences between groups (data not shown).

Our results demonstrate that, depending on its duration, stress exposure elicits opposite effects on the proinflammatory PGE_2_/anti-inflammatory 15d-PGJ_2 _balance in spinal cord of EAE-induced Dark Agouti rats. Exposure to 12 days of immobilisation stress induces a consistent increase in PGE_2_, MMP-9 over-expression and increased EAE clinical score. Conversely, chronically stressed (21 days) EAE-induced DA rats show increased 15d-PGJ_2 _levels, amelioration of clinical score, and reduced myelin loss, MMP-9 expression, MPO activity, and accumulation of neuroinflammatory mediators in the spinal cords of these animals. Further studies are needed to elucidate whether these changes in prostaglandins balance are sufficient to mediate the differences in clinical score and biochemical parameters here reported in the different temporal paradigms.

Supporting the existence of a causal interaction, some authors have established a relationship between acute relapse and PGE_2 _and determined the roles of mPGES-1 and PGE_2 _in EAE by the AA cascade-targeted lipidomics approach, and found that the PGE_2 _pathway is favoured and the PGD_2_, PGI_2_, and 5-Lipooxigenase pathways are attenuated. Correlation analysis imply that the AA-producing enzyme cPLA2 possibly passes more AA to COX than the 5-LO pathway in EAE, because the sequential actions of eicosanoid synthesizing enzymes are regulated spatially and temporally in the cells, the so-called functional coupling [42]. Likewise, PGH_2_, a common precursor of PGs, appears to be selectively consumed by the PGE_2 _pathway rather than the PGI_2 _and PGD_2 _pathways, thereby producing PGE_2 _in the spinal cords of EAE mice [43]. In fact, levels of proinflammatory/anti-inflammatory PGs have already been shown to be regulated by each other: previous studies have shown that 15d-PGJ_2 _is a potent blocker of mPGE synthase mRNA induction by LPS or IL-1β in macrophage-like differentiated U937 cells [44], fibroblasts and macrophages [45]. Similarly, we have previously demonstrated that administration of supraphysiological doses of 15d-PGJ_2 _can prevent the release of PGE_2 _and COX2 increased expression after stress exposure [24].

Some authors have directly implicated PGE_2 _in the pathophysiology of EAE and others have used 15d-PGJ_2 _as an anti-inflammatory agent against the disease. In this vein, PGE_2 _produced by microglia/macrophages may aggravate EAE by promoting differentiation of Th1 and expansion of Th17 cells [46], with a consequent increase of potentially deleterious proinflammatory cytokines, as has been demonstrated in other inflammatory scenarios [47,48].

On the other hand, PPARγ activation by the administration of 15d-PGJ_2 _ameliorates the pathophysiology of the disease, decreasing the clinical symptoms by reducing inflammation and blocking IL-12 dependent Th1 differentiation [30,49], and 15d-PGJ_2 _and retinoid × receptor ligands exert additive anti-inflammatory effects on another EAE model based on transgenic mice bearing the rearranged Va2.3;Vh8.2 gene encoding the TCR specific for the Ac1-11 peptide MBP [29]. Besides, 15d-PGJ_2 _is also effective in inhibiting production of cytotoxic molecules, including nitric oxide (NO), TNFα and interleukin 12, by microglial cells stimulated with interferon (IFN) γ and TNFα, molecules that have been implicated in pathogenesis of EAE and MS [28], by means of mechanisms that are PPARγ-independent. Regarding the inflammatory parameters analyzed in our studies, 15d-PGJ_2 _administration inhibits MMP-9, iNOS and COX-2 expression, MPO activity and MDA accumulation in inflammation-related pathologies such as restraint stress exposure, cerebral ischaemia, and colonic inflammation and dysfunction [24,50,51].

Here we demonstrate that shifting pathways towards anti-inflammatory prostaglandins by chronic stress exposure may actually be beneficial for the course of EAE, which is in accordance with the studies presented above. More importantly it would provide support to potential pharmacological modulation of PPARγ/15d-PGJ_2 _pathway or other anti-inflammatory strategies in MS.

Immobilization stress differentially modulates the progression and outcome of EAE, but the underlying biochemical mechanisms that explain the effects of brief exposure to stress aggravating EAE disease are not completely understood. In addition to increased levels of proinflammatory PGE_2 _that could modulate Th cell activity, the data obtained here suggest that subchronic stress could disrupt BBB integrity by enhancing MMP-9 expression (MMPs can influence expansion of the extracellular matrix of the BBB and loosening of intercellular tight junctions between endothelial cells through proteolytic degradation) and, as an increase in BBB permeability occurs prior to observable cellular infiltration in EAE [52], this may lead to an earlier appearance of clinical signs in stressed and inoculated animals. Chandler et al (2002) have already proposed that stress has a profound effect on the BBB, modulating EAE and MS. They hypothesized that while chronic stress may have inhibiting effects, acute stress may be disease-enhancing [9].

Results obtained from our different stress protocols indicate that physiological response to stress consists, in the case of this disease, also in a double, biphasic response: stress applied from the moment of inoculation with MOG provokes an earlier onset of disease, but longer exposure to stress makes the disease less severe. An adequate HPA response is necessary for correct control of disease; in fact, rat strains with a blunted HPA response are more vulnerable to EAE. However, elevated HPA activation in MS patients has been shown: in one study patients with MS had significantly higher plasma cortisol levels at baseline compared to matched controls. Despite this hypercortisolism, patients with MS showed normal, rather than blunted, plasma ACTH responses to ovine CRH, suggesting that the pathophysiology of hypercortisolism in MS is different from that in depression. Patients with MS also show blunted ACTH response to AVP stimulation, and normal cortisol response to high- and low-dose ACTH stimulation [53]. In the periphery, glucocorticoids inhibit innate and adaptative responses, thereby inhibiting lymphocyte proliferation and apoptosis of basophils and eosinophils, eliciting a redistribution of T cells and making dendritic cells stay in an immature state. Stress hormones have been implicated in reducing lymphocyte proliferation and NK cell activity, cytokine, and antibody production [54]. These facts turn out to be beneficial in the course of EAE disease [20]. Besides, it has been established that in EAE the HPA axis is activated in during clinical exacerbations, and that levels of corticosterone decrease as clinical signs remit [55]. However, there is emerging evidence that GC sensitivity of immune cells is impaired in MS [56]: while the HPA axis responds to inflammation during relapses [57], this response may be insufficient to control the inflammation

In our study, stress and EAE increased plasma levels of corticosterone and there were no differences between groups at the moment of sample extraction. However, it is to be noted that in the EAE+S group a daily glucocorticoid response was induced, as stress exposure started at the same time as administration of MOG and this was repeated until the day of sample extraction. Moreover, glucocorticoid receptor expression in spinal cord is lower in the EAE+S group at 12 days than in the control group, indicating a possible dysregulation of HPA feedback regulation (high levels of corticosterone not correlated with increased expression of GR) which indicates that hypofunction of the axis increases vulnerability and severity of EAE. It is to be noted that, although it has been proposed as the best animal model for MS, EAE has been recently criticised because numerous therapeutical approaches that showed promising results in this model in the end turned out to be either inefficient or in some cases harmful in human MS [58]. The results obtained from this animal model have to be considered carefully, but they may serve to understand the mechanisms and possible pharmacological modulation in MS and they clearly demonstrate an effect of stress exposure in the course of disease.

## Conclusions

In summary, immobilisation stress exposure paradigms differently modulate EAE pathophysiology at the clinical level and allow the identification of and dynamic regulation of possible biochemical pathways implicated in the degenerative process of the disease, opening new avenues for therapeutical strategies for MS (always considering the limitations of the EAE model). Further complementary studies are needed to elucidate the role of stress exposure in the aetiology and outcome of MS and the intrinsic mechanisms implicated.

## Competing interests

The authors declare that they have no competing interests.

## Authors' contributions

BGP-N contributed to acquisition of data, analysis and interpretation of data, conception and design, drafting the manuscript and revising it critically for important intellectual content; BGB contributed to analysis and interpretation of data, drafting the manuscript and revising it critically, JLMM contributed to contributed to analysis and interpretation of data, drafting the manuscript and revising it critically; and JCL contributed to conception and design, drafting the manuscript and revising it critically for important intellectual content. All authors have given final approval of the version to be published.
